# Outcomes and Predictors of Toxicity after Selective Internal Radiation Therapy Using Yttrium-90 Resin Microspheres for Unresectable Hepatocellular Carcinoma

**DOI:** 10.3389/fonc.2015.00292

**Published:** 2015-12-23

**Authors:** Andrew Gabrielson, Akemi Miller, Filip Banovac, Alexander Kim, Aiwu Ruth He, Keith Unger

**Affiliations:** ^1^Division of Hematology and Oncology, Lombardi Comprehensive Cancer Center, Georgetown University Hospital, Washington, DC, USA; ^2^Department of Interventional Radiology, Georgetown University Hospital, Washington, DC, USA; ^3^Department of Radiation Medicine, Georgetown University Hospital, Washington, DC, USA

**Keywords:** yttrium-90, SIRT, hepatocellular carcinoma, toxicity

## Abstract

**Purpose:**

We sought to report outcomes and toxicity in patients with hepatocellular carcinoma (HCC) who received resin yttrium-90 selective internal radiation therapy (^90^Y-SIRT) and to identify factors associated with declining liver function.

**Methods:**

Patients treated with ^90^Y-SIRT were retrospectively evaluated. Radiographic response was assessed using RECIST 1.1. Median liver progression-free survival (LPFS) and overall survival (OS) were calculated using the Kaplan–Meier method. Bivariate analysis was used to examine associations between change in Child-Pugh (CP) score/class and patient characteristics and treatment parameters.

**Results:**

Twenty-seven patients with unresectable HCC underwent SIRT, 52% were CP Class A, 48% were Class B, 11% were BCLC stage B, and 89% were stage C. Forty-four percent of patients had portal vein thrombus at baseline. One-third of patients received bilobar treatment. Median activity was 32.1 mCi (range 9.18–43.25) and median-­absorbed dose to the liver was 39.6 Gy (range 13.54–67.70). Median LPFS and OS were 2.5 and 11.7 months, respectively. Three-month disease control rate was 63 and 52% in the target lesions and whole liver, respectively. New onset or worsened from baseline clinical toxicities were confined to Grade 1–2 events. However, new or worsened Grade 3–4 laboratory toxicities occurred in 38% of patients at 3 months and 43% of patients at 6 months following SIRT (six had lymphocytopenia, three had hypoalbuminemia, and two had transaminasemia). After 3 months, six patients had worsened in CP score and five had worsened in class from baseline. After 6 months, four patients had worsened in CP score and one had worsened in class from baseline. Pretreatment bilirubinemia was associated with a 2+ increase in CP score within 3 months (*P* = 0.001) and 6 months (*P* = 0.039) of ^90^Y-SIRT. Pretreatment transaminasemia and bilirubinemia were associated with increased CP class within 3 months of SIRT (*P* = 0.021 and 0.009, respectively).

**Conclusion:**

^90^Y-SIRT was well-tolerated in patients with unresectable HCC, with no Grade 3–4 clinical toxicities. However, Grade 3–4 laboratory toxicities and worsened CP scores were more frequent. HCC patients with pretreatment bilirubinemia or transaminasemia may be at higher risk of experiencing a decline in liver function following ^90^Y-SIRT.

## Introduction

Hepatocellular carcinoma (HCC) is the sixth most common malignancy worldwide and the second most common cause of cancer-related mortality (1). Transplantation and surgical resection are potentially curative options but only 30–40% of the patients are amenable to curative treatment at diagnosis (2). As most patients are not eligible for curative treatment they must rely on systemic and locoregional therapies. Systemic therapies, such as sorafenib, a multi-targeted small molecule tyrosine kinase inhibitor, have demonstrated comparatively modest but still poor survival improvements of 2–3 months in patients with advanced HCC who are not candidates for transplant or resection (3). However, the response rate on sorafenib is <5% and all initially responding patients eventually develop resistance (3). Locoregional therapies for HCC can have a significant impact on the course of the disease, since many patients die of progressive disease (PD) within the liver. While external radiation therapy is an option for selected patients, this technique is often limited by the tolerance of the normal liver parenchyma.

Selective internal radiation therapy is emerging as an important locoregional modality in the treatment of metastatic and primary liver tumors. The treatment involves intra-arterial administration of resin or glass microspheres containing beta-emitting ^90^Y that preferentially accumulate in liver tumors and deliver cytotoxic doses of radiation (4). This technique exploits the liver’s unique vasculature system in which liver tumors derive up to 90% of their blood supply from the hepatic artery, while normal liver parenchyma is predominantly supplied by the portal venous system (5).

Multiple studies have demonstrated the feasibility and tolerability of ^90^Y SIRT for the treatment of liver metastasis and primary liver tumors (6–10). SIRT is often associated with mild toxicities in the first several days following treatment, including nausea, vomiting, fatigue, abdominal pain, and fever (8, 9). Serious adverse events, including radiation pneumonitis and gastric ulceration resulting from radiation applied to non-target tissues, are rare (10). While transient changes in liver function tests following SIRT are common, radiation-induced liver dysfunction (RILD) or liver failure is reported in <5% of patients (11). Classic RILD is defined as anicteric hepatomegaly, ascites, and elevated alkaline phosphatase (ALP) occurring 2 weeks to 4 months after radiation therapy (12).

Unlike patients with metastatic disease to the liver, many patients with HCC and cirrhosis have preexisting liver dysfunction, which may predispose them to toxicity following SIRT. The purpose of this study is to report outcomes and evaluate clinical and laboratory toxicities in patients with HCC treated with ^90^Y resin SIRT. In addition, we seek to identify predictive markers associated with the development of worsening hepatic function following SIRT.

## Materials and Methods

### Patient Cohort

Twenty-seven patients with primary, unresectable HCC treated with ^90^Y SIRT at the MedStar Georgetown University Hospital, Washington, DC, USA, between February 2012 and September 2014 were evaluated. A comprehensive review of imaging studies, survival outcomes, and clinical toxicities was performed, and data were collected retrospectively. A secure HIPAA-compliant database was constructed for the management of patient data. The MedStar/Georgetown Institutional Review Board approved the ethical, legal, and social implications of the study.

### Pretreatment Evaluation and Patient Selection

All patients underwent pretreatment evaluations, including clinical history and physical examination, as well as laboratory and imaging baseline assessments. Eligible patients had a biopsy confirmed or radiographic diagnosis of HCC that was deemed surgically unresectable due to tumor location and/or bulk, and not amenable to liver transplant. Patients had to be at least 18 years old, have an Eastern Cooperative Oncology Group (ECOG) score of 0 or 1, and have a life expectancy of >3 months. Patients were excluded for inadequate liver function (Grade 2+ ascites, serum albumin <3.0 g/dL, and total bilirubin >2.0 mg/dL), radiation exposure to the lungs of >30 Gy in a single fraction or 50 Gy in multiple administrations, and uncorrectable flow of ^90^Y microspheres to the gastrointestinal tract. The presence of portal vein thrombus and/or extrahepatic metastasis was not considered exclusionary.

### SIRT Planning and Procedure

All patients were treated with resin ^90^Y microspheres. The details for the procedure can be found in the 2013 version of the SIR-Spheres^®^ product insert and in Kennedy et al. (13, 14). A pretreatment diagnostic angiogram was performed in all patients, and specific extrahepatic vessels were coil embolized to prevent ^90^Y-microspheres from being distributed into visceral organs other than the liver during the SIRT procedure. Arteries that were actively sought and embolized included the gastroduodenal artery, the right gastric artery, the pancreaticoduodenal vessels, and any other relevant arteries depending on the patient-specific anatomy. A technetium-99m macroaggregated albumin (^99^m-Tc-MAA) SPECT scan was performed to calculate the lung shunt fraction, and a dose reduction was applied to maintain a lung radiation dose below 30 Gy. The prescribed activity of ­microspheres to be delivered was calculated using body surface area (BSA) method, which incorporates BSA, liver lobe volume, and percent tumor involvement of the lobe into the dose calculation (14). If stasis was achieved prior to full delivery of the prescribed dose of microspheres, the infusion was stopped and the dose delivered was recorded. A post-embolization Bremsstrahlung SPECT scan was performed to confirm the location of microsphere delivery.

### Toxicity Assessment

Patients typically underwent outpatient follow-up at 1 week, 1, and 3 months after ^90^Y SIRT, and then every 3 months thereafter until death. At these visits, physical examination and laboratory tests were performed. The following laboratory parameters were included in our analyses in order to assess laboratory toxicity: total bilirubin, ALP, aspartate aminotransferase (AST), alanine aminotransferase (ALT), albumin, hemoglobin (HGB), and lymphocytes. Blood samples taken up to 4 weeks prior to ^90^Y SIRT and at 3, 6 months, and 1 year during posttreatment follow-up were used for toxicity analysis. Clinical and laboratory toxicities were graded according to the Common Terminology Criteria for Adverse Events (CTCAE) v4.03. If baseline and follow-up laboratory data were not available for any particular patient in the chosen cohort, this patient was excluded from the laboratory toxicity assessment. Clinical toxicity assessment was based on the reporting in patients’ charts of periprocedural complications, treatment-related symptoms (CTCAE Grade 1–2), and serious adverse events (CTCAE Grade 3–4). Laboratory and clinical toxicities noted following radiographically confirmed progression of disease or subsequent locoregional therapy were excluded from analyses.

Child-Pugh (CP) score was used to assess changes in liver function after SIRT. An increase in CP score by two or more points was deemed to be a clinically relevant indicator of decline in liver function following radiotherapy in one prior analysis (15). For data analysis purposes, changes in liver function were expressed as a binary value at 3 and 6 months post-SIRT: patients with a 2+ increase in CP score from baseline were given a 1 at each follow-up point; those with a score increase of <2 were given a 0. The observation of clinical/laboratory toxicities and a 2+ point increase in CP score in the absence of HCC progression and subsequent locoregional therapy were considered treatment related and were the focus of our analyses.

### SIRT Response Assessment

Baseline imaging of the liver was performed using either CT or MRI techniques. The choice of follow-up imaging methodology was the same as that used for baseline imaging and occurred at approximately 3 posttreatment. Response assessment was performed in accordance with the Modified Response Evaluation Criteria in Solid Tumors version 1.1 (RECIST 1.1) on the level of target lesions and whole liver (includes non-target and new lesions) at 3 months after SIRT.

### Statistical Analysis

Liver progression-free survival (LPFS) and overall survival (OS) were calculated using Kaplan–Meier survival analyses and were calculated from the first ^90^Y SIRT treatment. Bivariate analyses were used to examine the association of CP score/class with patient characteristics and treatment parameters. Statistical analyses were performed using SPSS Statistics 21.0 for Windows (IBM SPSS, Chicago, IL, USA); *P* < 0.05 was considered statistically significant.

## Results

### Patient Demographics

The demographic and baseline characteristics of study patients are shown in Table 1. The mean age was 62.2 (range, 39–85) and the majority (74%) were male. At baseline, most patients (85%) had an ECOG performance status of 0 or 1, and only 30% had extrahepatic metastasis. Twelve patients (44%) had tumor portal vein thrombus, according to baseline scans, and pretreatment liver function assessment showed an equal number of patients with CP A (52%) and CP B disease (48%). Pretreatment BCLC staging identified 3 patients (11%) as BCLC stage B and 24 patients (89%) as BCLC stage C. The most common comorbidities were hypertension (93%, *n* = 25), ascites (70%, *n* = 19), liver cirrhosis (70%, *n* = 19), hepatitis C viral infection (63%, *n* = 17), and diabetes mellitus type II (55%, *n* = 15). Twenty-three patients (85%) had received prior systemic therapy. Twenty-one patients (78%) were treated with sorafenib, one patient (4%) was treated with combination 5-flurouracil and oxaliplatin, and one patient (4%) was treated with combination gemcitabine and cisplatin prior to SIRT.

**Table 1:** **Baseline characteristics**.

Characteristic	Patients (%)
Age (median, min–max)	62.2 (39–85)
Sex [*n* (%)]	
Female	7 (26)
Male	20 (74)
ECOG performance status	
0	6 (22)
1	17 (63)
2	2 (7)
Unreported	2 (7)
Comorbidities	
Hypertension	25 (93)
Ascites	19 (70)
Liver cirrhosis	19 (70)
Hepatitis C infection	17 (63)
Diabetes mellitus type II	15 (55)
Coronary artery disease	5 (19)
Hepatitis B infection	5 (19)
Child-Pugh score	
5A–6A	14 (52)
7B–9B	13 (48)
BCLC stage	
B	3 (11)
C	24 (89)
Extrahepatic metastasis	
Yes	8 (30)
No	18 (66)
Unreported	1 (4)
Portal vein thrombus	
Yes	12 (44)
No	15 (56)
Prior systemic treatment	
Sorafenib	21 (78)
5-Flurouracil + oxaliplatin	1 (4)
Gemcitabine + cisplatin	1 (4)
Subsequent locoregional therapy	
TACE	9 (33)

### Treatment Details

Treatment information is shown in Table 2. One-third of patients (*n* = 9) received bilobar treatment, with no patients receiving treatment of both lobes on the same day. The mean lung shunt fraction was 6.52% and six patients (22%) received a reduced dose at the discretion of the treating physicians. The median administered radioactivity was 32.1 mCi (range 9.18–43.25) with an estimated median liver-absorbed dose of 39.6 Gy (range 13.54–67.7). No periprocedural complications occurred during ^90^Y microsphere administration. There were no instances of extrahepatic deposition of radioactivity based on posttreatment bremsstrahlung scintigraphy.

**Table 2:** **SIRT treatment details**.

Treatment variable	
99mTc-MAA lung shunt fraction (mean)	6.52%
Dose reduction required in “*n*” patients	6 (20%)
Mean ^90^Y administered activity (MBq)	1185 ± 340
Mean ^90^Y liver-absorbed dose (Gy)	39.6 ± 13.1
Number of patients with concurrent ^90^Y treatment and chemotherapy (*n*)	7 (23%)
Patients receiving bilobar treatment (*n*)	9 (30%)

### Toxicities

**Figure 1:**
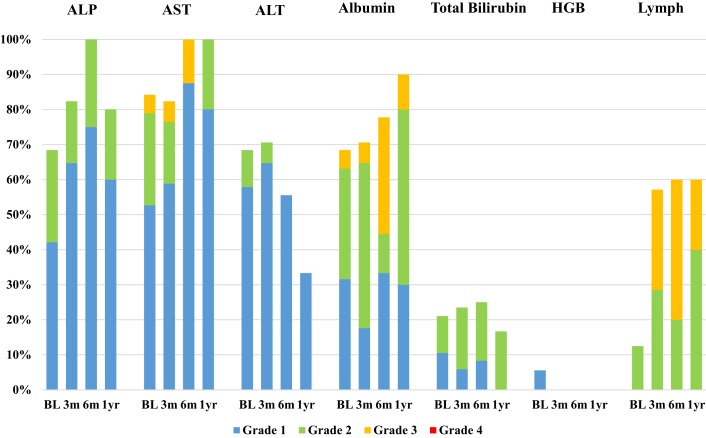
**Bar chart demonstrating the incidence of laboratory toxicities at baseline visit (BL) and 3, 6 months, and 1 year after SIRT**. Laboratory parameters included alkaline phosphatase (ALP), aspartate aminotransferase (AST), alanine aminotransferase (ALT), albumin, total bilirubin, hemoglobin (HGB), and lymphocytes (Lymph). Toxicities were classified by CTCAE Grade: blue = Grade 1, green = Grade 2, yellow = Grade 3, and red = Grade 4.

Median follow-up time was 10.1 months (range 0.6–29.6). Twenty-three patients were included in the toxicity analysis; four patients (15%) in our cohort were excluded due to incomplete laboratory data or inappropriate timing of laboratory follow-up for our study. Laboratory toxicities occurring at 3, 6 months, and 1 year after SIRT were classified by CTCAE Grade and summarized in Figure 1. The number of patients at risk of developing Grade 3 or 4 laboratory toxicities at 3 and 6 months after SIRT was 16 and 7, respectively, after excluding patients who had PD in the liver or received additional liver directed therapy. At 3 months after SIRT, 6 out of 16 patients at risk (38%) suffered new onset or worsened from baseline Grade 3 or 4 laboratory toxicities: 4 patients (25%) had lymphocytopenia, 1 patient (6%) had hypoalbuminemia, and 1 patient had transaminasemia. At 6 months after SIRT, three out of seven patients at risk (43%) suffered new onset or worsened from baseline Grade 3 or 4 laboratory toxicities: two patients (29%) had lymphocytopenia, and one patient had hypoalbuminemia. All other laboratory toxicities were limited to Grade 1–2 events.

Clinical toxicities occurring within 1 year of SIRT are summarized in Table 3. New onset or worsened from baseline clinical toxicities were confined to Grade 1 or 2 events. Many of these symptoms were associated with post-embolization syndrome, which resolved before the first 3 months of follow-up. No Grade 3 or 4 clinical toxicities were observed after yttrium-90 selective internal radiation therapy (^90^Y-SIRT) and no radiation-induced complications, such as gastrointestinal ulceration, pulmonary toxicity, pancreatitis, or radiation pneumonitis, were observed.

**Table 3:** **Clinical toxicities following SIRT**.

Grade 1 or 2 adverse events	Patients (%)
Abdominal pain	23 (85)
Fatigue	22 (81)
Ascites	18 (67)
Nausea	12 (44)
Diarrhea	10 (37)
Vomiting	7 (26)
Hepatic encephalopathy	5 (19)
Fever	3 (11)
Jaundice	3 (11)

### Liver Function Assessment

After 3 months, 6 out of 16 patients at risk (38%) had experienced an increase in CP score and 5 (32%) showed an increase in CP class. After 6 months of follow-up, four out of seven patients at risk (57%) had an increased CP score and one (14%) showed an increase in CP class. An increase of at least 2 points in the CP score was noted in nine patients (39%) during the 1-year follow-up period. Among these nine patients, two patients (22%) recovered from the CP score increase within 6 months of SIRT treatment.

### Tumor Response and Survival

Tumor response to therapy at both the target lesion and whole liver level were analyzed according to RECIST 1.1 criteria at 3 months (range, 1.5–4) following ^90^Y SIRT (Table 4). Three patients (11%) were excluded from radiographic analysis due to lack of evaluable imaging or change in imaging modality. At 3 months posttreatment, the disease control rate [complete response (CR) + partial response (PR) + stable disease (SD)] was 63% for target lesions and 52% for the whole liver. Seven patients (26%) had PD for the target lesions and 10 patients (37%) had PD of the whole liver after 3 months posttreatment. Nine patients (33%) received subsequent locoregional therapy (trans-arterial chemoembolization) following SIRT.

**Table 4:** **Tumor response to SIRT at the target lesion and whole-liver level after 3 months of follow-up**.

	Target lesions	Whole liver
Complete response	0 (0%)	0 (0%)
Partial response	1 (4%)	1 (4%)
Stable disease	16 (59%)	13 (48%)
Progressive disease	7 (26%)	10 (37%)
Not evaluable	3 (11%)	3 (11%)
Disease control rate	63%	52%

**Figure 2:**
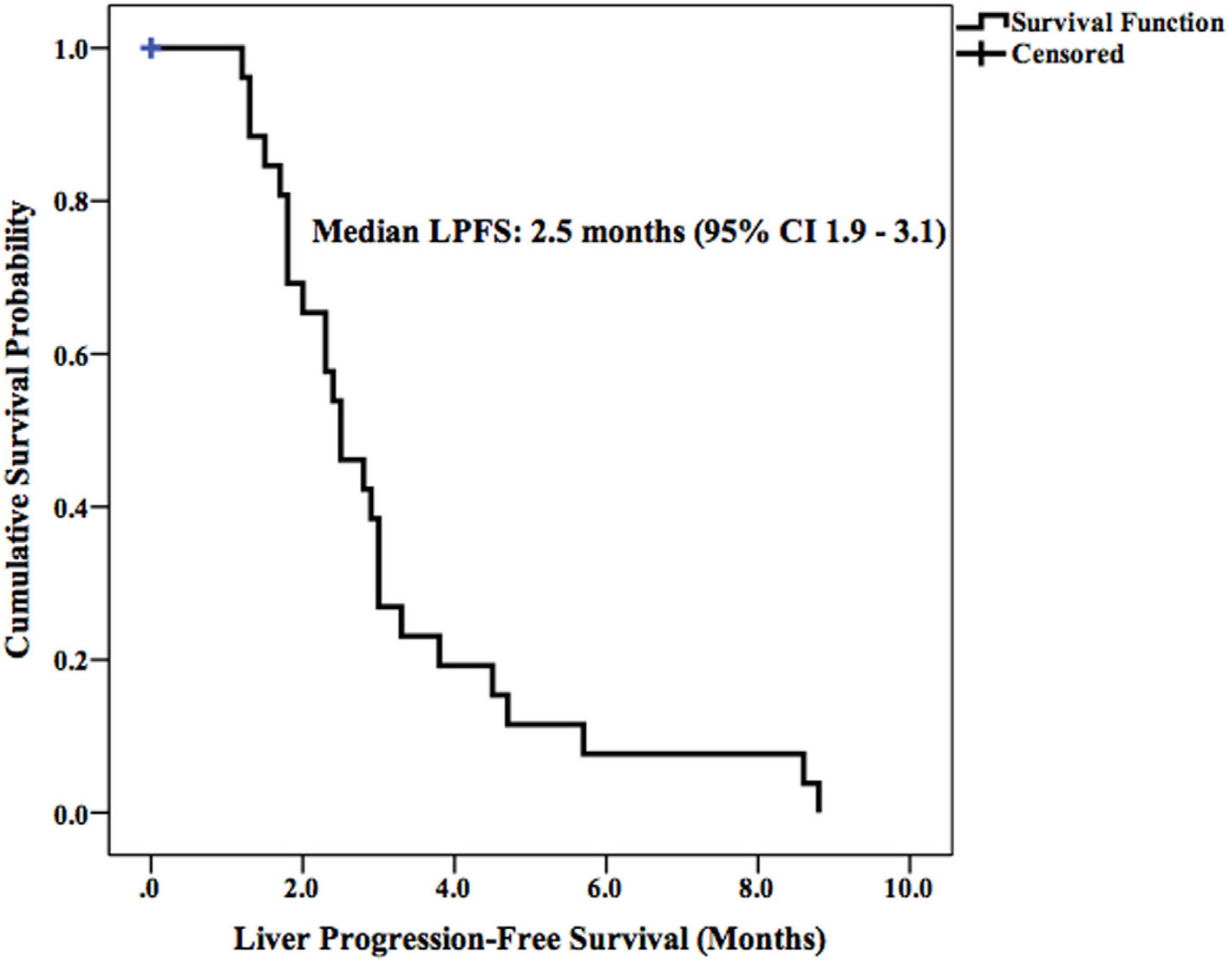
**Kaplan–Meier curve of LPFS in patients receiving SIRT**.

**Figure 3:**
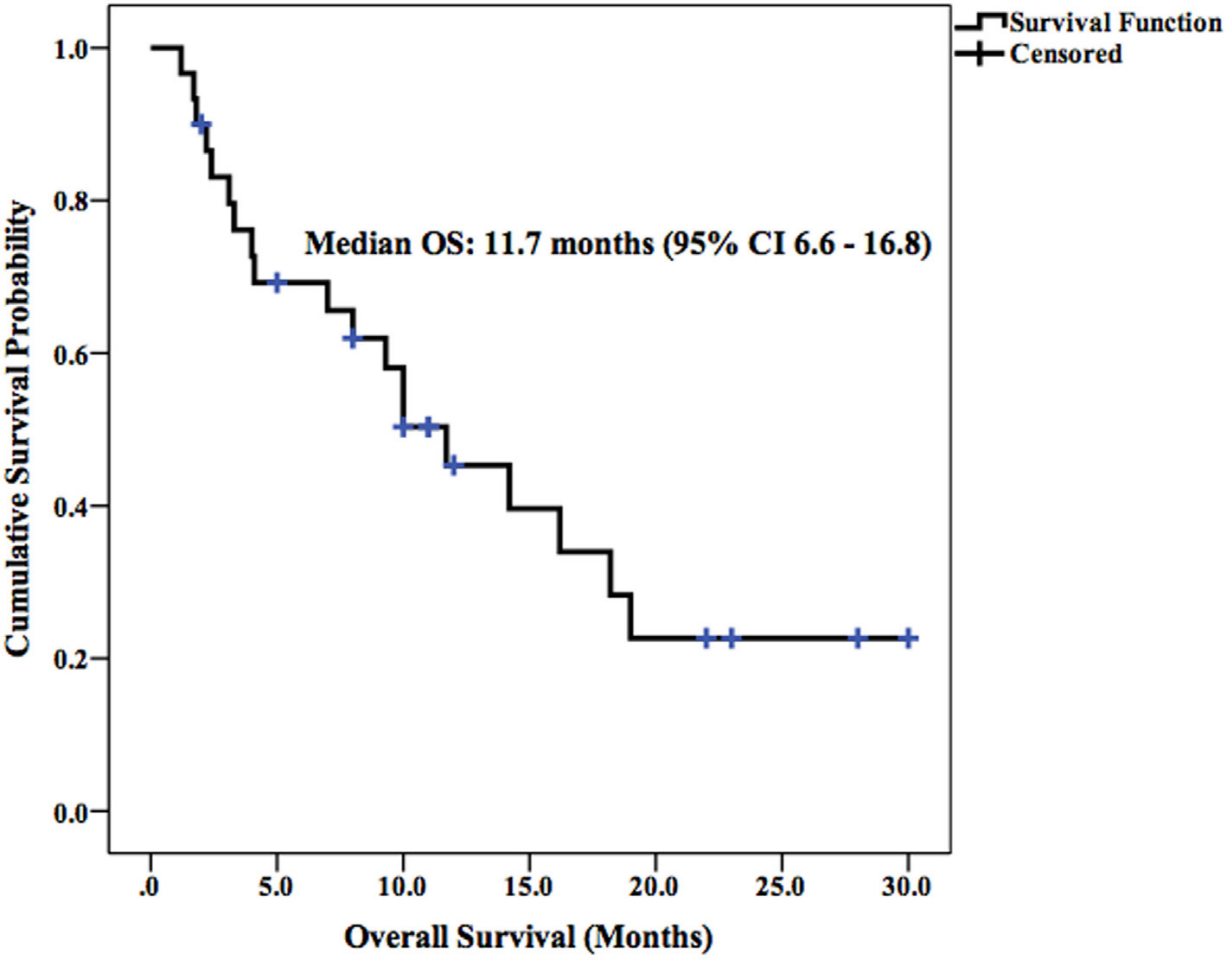
**Kaplan–Meier curve of OS in patients receiving SIRT**.

**Figure 4:**
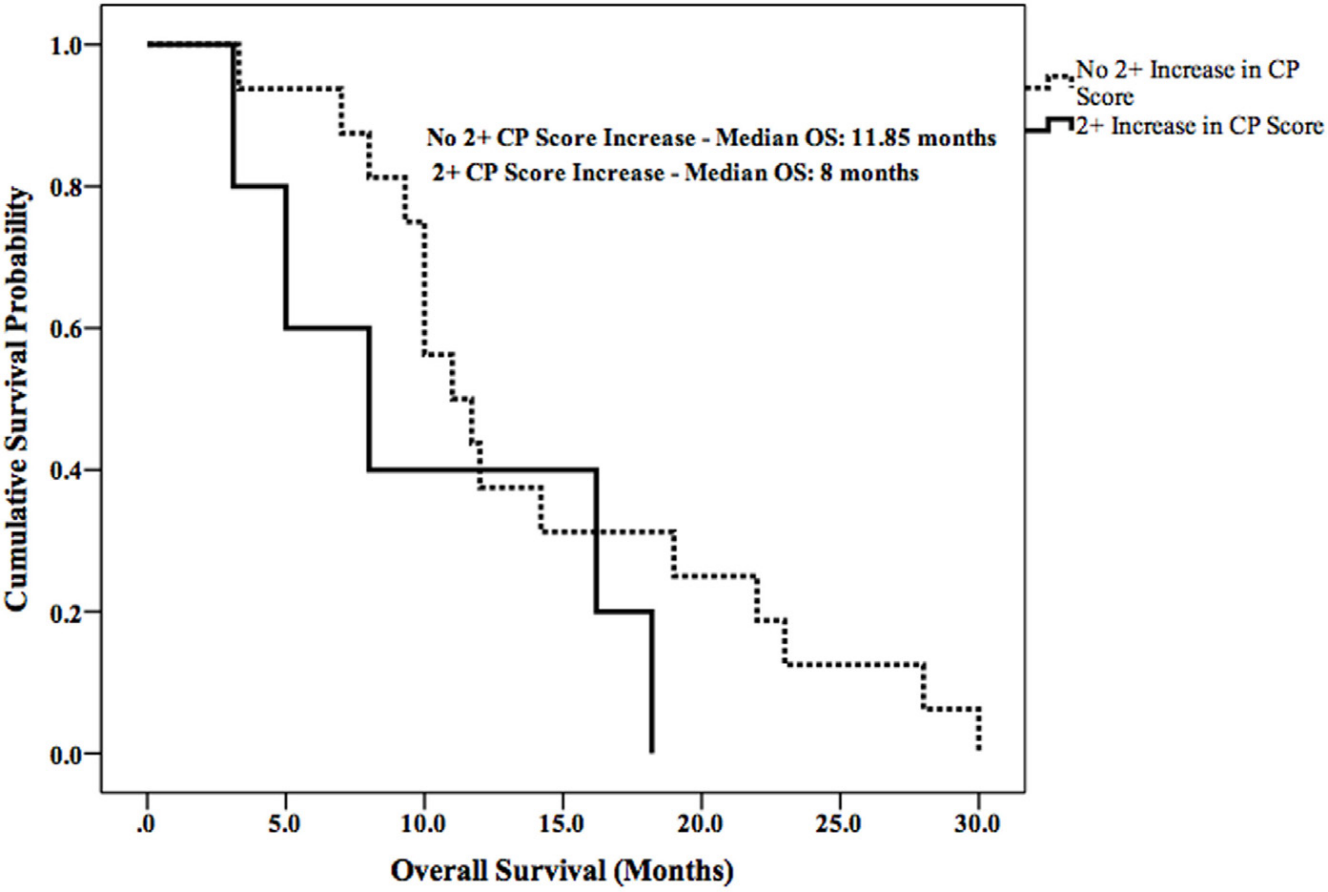
**Kaplan–Meier curves comparing OS in patients with or without a 2+ increase in CP score**.

**Figure 5:**
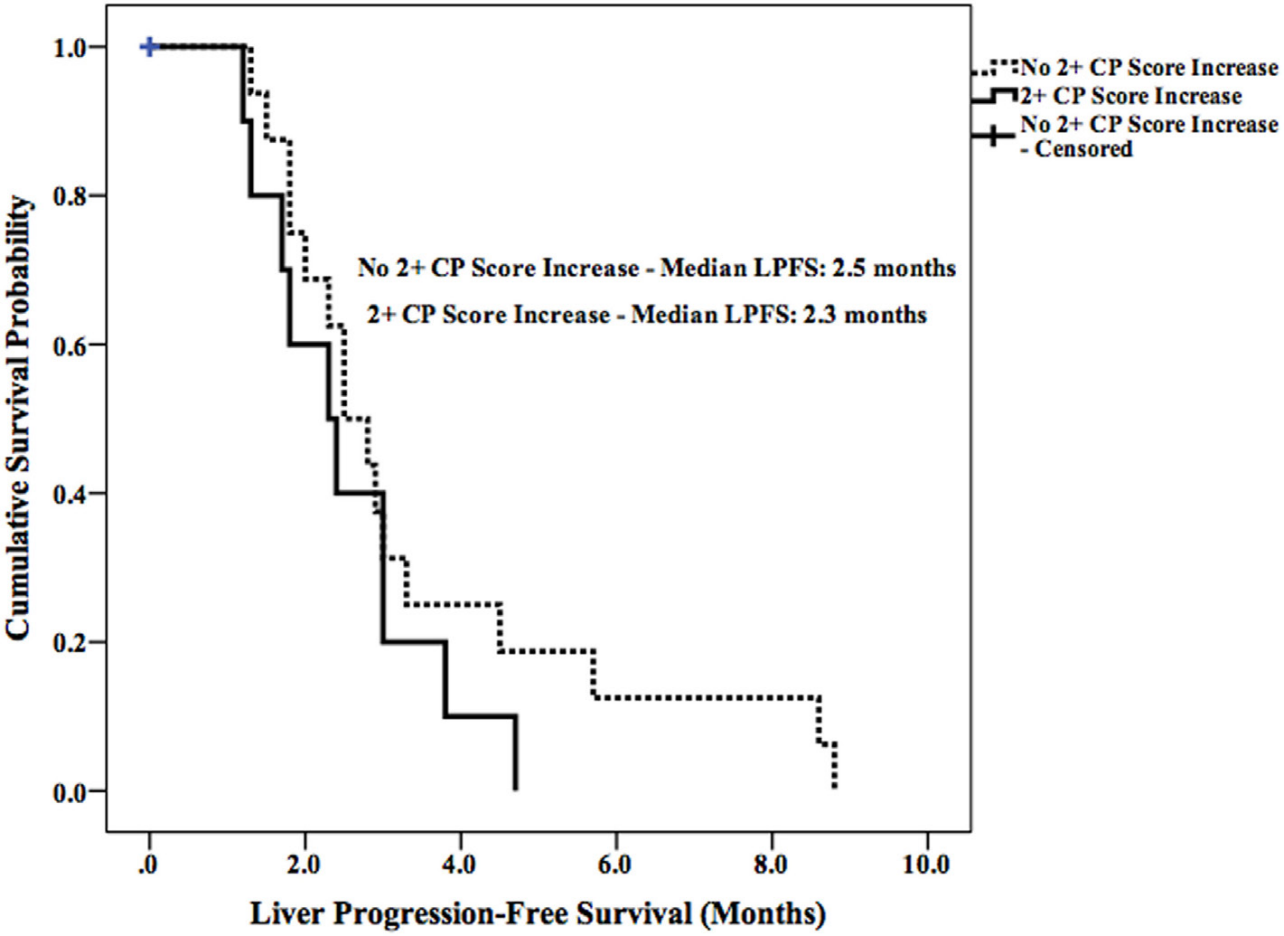
**Kaplan–Meier curves comparing LPFS in patients with or without a 2+ increase**.

Median LPFS and OS for the entire cohort were 2.5 months (95% CI 1.9–3.1) (Figure 2) and 11.7 months (95% CI 6.6–16.8) (Figure 3), respectively. In the absence of a 2+ increase in CP score within the first 6 months of follow-up, median LPFS was 2.5 months (95% CI 1.8–3.2) and median OS was 11.85 months (95% CI 7.7–14.3); patients who experienced a 2+ increase in CP score within the first 6 months of follow-up had a median LPFS of 2.3 months (95% CI 1.3–3.2) and median OS of 8 months (95% CI 1.6–14.4) (Figures 4 and 5).

### Association Between Laboratory/Treatment Parameters and Decline in Liver Function Following SIRT

Bivariate analyses were used to assess the association between baseline laboratory/treatment parameters and postoperative decline in liver function, as defined by a 2+ increase in CP score or 1+ increase in CP class. Bivariate analyses showed that pretreatment bilirubinemia was associated with a 2+ increase in CP score within 3 months (*P* = 0.001) and 6 months (*P* = 0.039) of SIRT. Pretreatment bilirubinemia and transaminasemia were associated with an increase in CP class within 3 months of SIRT (*P* = 0.021, 0.028, and 0.009, respectively). The following pretreatment factors were not significantly related with a decline in posttreatment liver function: ALP, albumin, HGB, CP class, model for end-stage liver disease (MELD) score, BCLC stage, portal vein thrombus at baseline, radioactivity, dose, and bilobar treatment.

## Discussion

Hepatocellular carcinoma is one of the most common forms of cancer worldwide, remaining the second leading cause of cancer-related death in men and the sixth leading cause in women (16). For many HCC patients, curative resection or transplantation is not an option due to tumor bulk or the presence of metastatic disease. Locoregional therapies, such as ^90^Y-SIRT, are a viable alternative method of reducing tumor burden, prolonging OS, and improving quality of life.

Our study has demonstrated that resin ^90^Y-SIRT is a well-tolerated treatment option for patients with primary unresectable HCC. Similar to previously published studies, clinical toxicities in the current study were limited to Grade 1 and 2 events, primarily those associated with post-embolization syndrome (17–19). The majority of these symptoms resolved within the first 3 months of follow-up after SIRT. Particularly deleterious clinical toxicities, such as gastrointestinal ulceration and radiation pneumonitis, were not observed in this study, likely due to careful patient selection and pretreatment diagnostic work-up. The other main safety consideration in this SIRT cohort was hepatotoxicity due to unintentional but unavoidable irradiation of benign liver tissue. Previous studies demonstrated that up to 70% of patients develop low-grade (Grade 1–2) abnormal liver function tests following SIRT (20–22), and the incidence of high-grade (Grade 3–4) laboratory toxicities has been reported to range from 0 to 38% (7, 20, 23, 24). In the current study, 65% (*n* = 15) and 39% (*n* = 9) of patients experienced Grade 1–2 and Grade 3 laboratory toxicities, respectively, within 6 months of follow-up. The most frequent Grade 3 toxicities were lymphocytopenia and hypoalbuminemia. No Grade 4 toxicities were observed.

According to recent studies, 3-month disease control rates following ^90^Y-SIRT in patients with advanced HCC range from 56 to 100% when evaluated using RECIST criteria (9, 25–27). The tumor response in the current study yielded comparable results: 3-month disease control rate was 63% in the target lesions and 52% in the whole liver. The median LPFS (2.5 months) and OS (11.7 months) observed in this study are comparable with the LPFS and OS observed in a number of studies examining the clinical benefit of ^90^Y-SIRT in patients with advanced HCC (28–32).

The risk of liver toxicity is an important consideration following SIRT (17, 33–35). Previous studies have attempted to identify radiographic and dosimetric predictors of liver toxicity; the percentage of liver volume receiving more than 30 Gy, percentage of lung volume receiving more than 20 Gy, pretreatment hepatopulmonary shunt fraction, and pretreatment liver cirrhosis have all been independently associated with liver toxicity following SIRT (36–42). A recent report demonstrated that a 2+ change in CP score is a clinically important indicator of decline in liver function and survival in patients with unresectable HCC who have received radiotherapy (15). This finding is supported by the survival data in our current study. Patients who did not develop a 2+ increase in CP score had prolonged LPFS (0.2 months longer) and OS (3.4 months longer) when compared with patients who did.

It has been suggested that HGB level prior to SIRT may be an important laboratory marker for tumor response and liver function in patients with liver metastases (43). To date, no studies have demonstrated an association between pretreatment laboratory values and liver toxicity following SIRT in primary HCC. In order to identify predictive markers of liver toxicity following SIRT in our cohort, patient baseline laboratory values and treatment parameters were correlated with the observed changes in CP score and class. Our analysis revealed a statistically significant association between pretreatment bilirubinemia and a 2+ increase in CP score within 3 and 6 months post-SIRT. Additionally, pretreatment bilirubinemia and transaminasemia were associated with a 1+ increase in CP class within 3 months of SIRT.

The most important limitations of this study were its retrospective design and the small number of patients (*n* = 23) involved due to the requirement of complete laboratory and clinical follow-up data. Additionally, determining the cause of liver dysfunction in this patient population is challenging. We excluded patients from the laboratory analysis following disease progression in the liver, which could result in underestimating the incidence of post-treatment liver toxicity. However, progression of cirrhosis independent of SIRT could overestimate the incidence of liver toxicity, as 48% of patients were CP class B in this study. Another limitation of this study is the focus on resin microspheres (SIR-Spheres). Glass microspheres (Therasphere) have been shown to have similar clinical and laboratory toxicities when compared to resin microspheres (44, 45). However, few studies have examined changes in CP score following SIRT using glass microspheres. Prospective studies utilizing a larger cohort may be warranted to validate the results of this study in both glass and resin microspheres.

Yttrium-90 selective internal radiation therapy was well tolerated in patients with unresectable HCC and resulted in encouraging disease control rates. There were no Grade 3–4 clinical toxicities; however, Grade 3–4 laboratory toxicities and worsened CP scores were more frequent. HCC patients with pretreatment bilirubinemia or transaminasemia were at higher risk of experiencing a decline in liver function following SIRT. Future research is required to optimize patient selection and to validate risk factors for liver toxicity.

## Author Contributions

AG – carried out medical record data mining, data analysis, and writing of the manuscript. AM – carried out radiographic analysis and provided manuscript support. AH – treated patients in the medical oncology setting, assisted in patient selection for ^90^Y-SIRT. FB, AK, and KU – carried out ^90^Y-SIRT procedures and provided oversight of study design.

## Conflict of Interest Statement

The authors declare that the research was conducted in the absence of any commercial or financial relationships that could be construed as a potential conflict of interest.
